# Supplementary material for: Resolving the full spectrum of human genome variation using Linked-Reads
Source: Genome Res. 2019 Apr;29(4):635–45. doi: 10.1101/gr.234443.118 (PMC6442396; doi:10.1101/gr.234443.118)
Supplement: Supplemental Material [file supp_gr.234443.118_Supplemental_longranger-2.2.zip › longranger-2.2.tar.bz2!/lr2.2-public/lib/stan/cmdstan/stan/doc/api/html/binomial_8hpp_source.html]

Stan: src/stan/prob/distributions/univariate/discrete/binomial.hpp Source File


|  |  |
| --- | --- |
|  | Stan  2.5.0  probability, sampling & optimization |


- Main Page
- Related Pages
- Namespaces
- Classes
- Files

- File List
- File Members

All Classes Namespaces Files Functions Variables Typedefs Enumerations Enumerator Friends Macros Pages

- src
- stan
- prob
- distributions
- univariate
- discrete

binomial.hpp

Go to the documentation of this file.

1 #ifndef STAN\_\_PROB\_\_DISTRIBUTIONS\_\_UNIVARIATE\_\_DISCRETE\_\_BINOMIAL\_HPP

2 #define STAN\_\_PROB\_\_DISTRIBUTIONS\_\_UNIVARIATE\_\_DISCRETE\_\_BINOMIAL\_HPP

3

4 #include <boost/random/binomial\_distribution.hpp>

5 #include <boost/random/variate\_generator.hpp>

6

7 #include <stan/agrad/partials\_vari.hpp>

8 #include <stan/math/error\_handling.hpp>

9 #include <stan/math/constants.hpp>

10 #include <stan/math/functions/inv\_logit.hpp>

11 #include <stan/math/functions/log1m.hpp>

12 #include <stan/math/functions/log\_inv\_logit.hpp>

13 #include <stan/math/functions/multiply\_log.hpp>

14 #include <stan/math/functions/value\_of.hpp>

15 #include <stan/meta/traits.hpp>

16 #include <stan/prob/traits.hpp>

17 #include <stan/prob/constants.hpp>

18

19 #include <stan/math/functions/binomial\_coefficient\_log.hpp>

20

21 namespace stan {

22

23  namespace prob {

24

25  // Binomial(n|N,theta) [N >= 0; 0 <= n <= N; 0 <= theta <= 1]

26  template <bool propto,

27  typename T\_n,

28  typename T\_N,

29  typename T\_prob>

30  typename return\_type<T\_prob>::type

31  binomial\_log(const T\_n& n,

32  const T\_N& N,

33  const T\_prob& theta) {

34

35  static const char\* function = "stan::prob::binomial\_log(%1%)";

36

37  using stan::math::check\_finite;

38  using stan::math::check\_bounded;

39  using stan::math::check\_nonnegative;

40  using stan::math::value\_of;

41  using stan::math::check\_consistent\_sizes;

42  using stan::prob::include\_summand;

43

44  // check if any vectors are zero length

45  if (!(stan::length(n)

46  && stan::length(N)

47  && stan::length(theta)))

48  return 0.0;

49

50  double logp = 0;

51  check\_bounded(function, n, 0, N,

52  "Successes variable",

53  &logp);

54  check\_nonnegative(function, N,

55  "Population size parameter",

56  &logp);

57  check\_finite(function, theta,

58  "Probability parameter",

59  &logp);

60  check\_bounded(function, theta, 0.0, 1.0,

61  "Probability parameter",

62  &logp);

63  check\_consistent\_sizes(function,

64  n,N,theta,

65  "Successes variable",

66  "Population size parameter",

67  "Probability parameter",

68  &logp);

69

70

71  // check if no variables are involved and prop-to

72  if (!include\_summand<propto,T\_prob>::value)

73  return 0.0;

74

75  // set up template expressions wrapping scalars into vector views

76  VectorView<const T\_n> n\_vec(n);

77  VectorView<const T\_N> N\_vec(N);

78  VectorView<const T\_prob> theta\_vec(theta);

79  size\_t size = max\_size(n, N, theta);

80

81  agrad::OperandsAndPartials<T\_prob> operands\_and\_partials(theta);

82

83  using stan::math::multiply\_log;

84  using stan::math::binomial\_coefficient\_log;

85  using stan::math::log1m;

86

87  if (include\_summand<propto>::value)

88  for (size\_t i = 0; i < size; ++i)

89  logp += binomial\_coefficient\_log(N\_vec[i],n\_vec[i]);

90

91  DoubleVectorView<true,is\_vector<T\_prob>::value> log1m\_theta(length(theta));

92  for (size\_t i = 0; i < length(theta); ++i)

93  log1m\_theta[i] = log1m(value\_of(theta\_vec[i]));

94

95  // no test for include\_summand because return if not live

96  for (size\_t i = 0; i < size; ++i)

97  logp += multiply\_log(n\_vec[i],value\_of(theta\_vec[i]))

98  + (N\_vec[i] - n\_vec[i]) \* log1m\_theta[i];

99

100  if (length(theta) == 1) {

101  double temp1 = 0;

102  double temp2 = 0;

103  for (size\_t i = 0; i < size; ++i) {

104  temp1 += n\_vec[i];

105  temp2 += N\_vec[i] - n\_vec[i];

106  }

107  if (!is\_constant\_struct<T\_prob>::value) {

108  operands\_and\_partials.d\_x1[0]

109  += temp1 / value\_of(theta\_vec[0])

110  - temp2 / (1.0 - value\_of(theta\_vec[0]));

111  }

112  } else {

113  if (!is\_constant\_struct<T\_prob>::value) {

114  for (size\_t i = 0; i < size; ++i)

115  operands\_and\_partials.d\_x1[i]

116  += n\_vec[i] / value\_of(theta\_vec[i])

117  - (N\_vec[i] - n\_vec[i]) / (1.0 - value\_of(theta\_vec[i]));

118  }

119  }

120

121  return operands\_and\_partials.to\_var(logp);

122  }

123

124  template <typename T\_n,

125  typename T\_N,

126  typename T\_prob>

127  inline

128  typename return\_type<T\_prob>::type

129  binomial\_log(const T\_n& n,

130  const T\_N& N,

131  const T\_prob& theta) {

132  return binomial\_log<false>(n,N,theta);

133  }

134

135  // BinomialLogit(n|N,alpha) [N >= 0; 0 <= n <= N]

136  // BinomialLogit(n|N,alpha) = Binomial(n|N,inv\_logit(alpha))

137  template <bool propto,

138  typename T\_n,

139  typename T\_N,

140  typename T\_prob>

141  typename return\_type<T\_prob>::type

142  binomial\_logit\_log(const T\_n& n,

143  const T\_N& N,

144  const T\_prob& alpha) {

145

146  static const char\* function = "stan::prob::binomial\_logit\_log(%1%)";

147

148  using stan::math::check\_finite;

149  using stan::math::check\_bounded;

150  using stan::math::check\_nonnegative;

151  using stan::math::value\_of;

152  using stan::math::check\_consistent\_sizes;

153  using stan::prob::include\_summand;

154

155  // check if any vectors are zero length

156  if (!(stan::length(n)

157  && stan::length(N)

158  && stan::length(alpha)))

159  return 0.0;

160

161  double logp = 0;

162  check\_bounded(function, n, 0, N,

163  "Successes variable",

164  &logp);

165  check\_nonnegative(function, N,

166  "Population size parameter",

167  &logp);

168  check\_finite(function, alpha,

169  "Probability parameter",

170  &logp);

171  check\_consistent\_sizes(function,

172  n,N,alpha,

173  "Successes variable",

174  "Population size parameter",

175  "Probability parameter",

176  &logp);

177

178  // check if no variables are involved and prop-to

179  if (!include\_summand<propto,T\_prob>::value)

180  return 0.0;

181

182  // set up template expressions wrapping scalars into vector views

183  VectorView<const T\_n> n\_vec(n);

184  VectorView<const T\_N> N\_vec(N);

185  VectorView<const T\_prob> alpha\_vec(alpha);

186  size\_t size = max\_size(n, N, alpha);

187

188  agrad::OperandsAndPartials<T\_prob> operands\_and\_partials(alpha);

189

190  using stan::math::binomial\_coefficient\_log;

191  using stan::math::log\_inv\_logit;

192  using stan::math::inv\_logit;

193

194  if (include\_summand<propto>::value)

195  for (size\_t i = 0; i < size; ++i)

196  logp += binomial\_coefficient\_log(N\_vec[i],n\_vec[i]);

197

198  DoubleVectorView<true,is\_vector<T\_prob>::value> log\_inv\_logit\_alpha(length(alpha));

199  for (size\_t i = 0; i < length(alpha); ++i)

200  log\_inv\_logit\_alpha[i] = log\_inv\_logit(value\_of(alpha\_vec[i]));

201

202  DoubleVectorView<true,is\_vector<T\_prob>::value> log\_inv\_logit\_neg\_alpha(length(alpha));

203  for (size\_t i = 0; i < length(alpha); ++i)

204  log\_inv\_logit\_neg\_alpha[i] = log\_inv\_logit(-value\_of(alpha\_vec[i]));

205

206  for (size\_t i = 0; i < size; ++i)

207  logp += n\_vec[i] \* log\_inv\_logit\_alpha[i]

208  + (N\_vec[i] - n\_vec[i]) \* log\_inv\_logit\_neg\_alpha[i];

209

210  if (length(alpha) == 1) {

211  double temp1 = 0;

212  double temp2 = 0;

213  for (size\_t i = 0; i < size; ++i) {

214  temp1 += n\_vec[i];

215  temp2 += N\_vec[i] - n\_vec[i];

216  }

217  if (!is\_constant\_struct<T\_prob>::value) {

218  operands\_and\_partials.d\_x1[0]

219  += temp1 \* inv\_logit(-value\_of(alpha\_vec[0]))

220  - temp2 \* inv\_logit(value\_of(alpha\_vec[0]));

221  }

222  } else {

223  if (!is\_constant\_struct<T\_prob>::value) {

224  for (size\_t i = 0; i < size; ++i)

225  operands\_and\_partials.d\_x1[i]

226  += n\_vec[i] \* inv\_logit(-value\_of(alpha\_vec[i]))

227  - (N\_vec[i] - n\_vec[i]) \* inv\_logit(value\_of(alpha\_vec[i]));

228  }

229  }

230

231  return operands\_and\_partials.to\_var(logp);

232  }

233

234  template <typename T\_n,

235  typename T\_N,

236  typename T\_prob>

237  inline

238  typename return\_type<T\_prob>::type

239  binomial\_logit\_log(const T\_n& n,

240  const T\_N& N,

241  const T\_prob& alpha) {

242  return binomial\_logit\_log<false>(n,N,alpha);

243  }

244

245

246  // Binomial CDF

247  template <typename T\_n, typename T\_N, typename T\_prob>

248  typename return\_type<T\_prob>::type

249  binomial\_cdf(const T\_n& n, const T\_N& N, const T\_prob& theta) {

250

251  static const char\* function = "stan::prob::binomial\_cdf(%1%)";

252

253  using stan::math::check\_finite;

254  using stan::math::check\_bounded;

255  using stan::math::check\_nonnegative;

256  using stan::math::value\_of;

257  using stan::math::check\_consistent\_sizes;

258  using stan::prob::include\_summand;

259

260  // Ensure non-zero arguments lenghts

261  if (!(stan::length(n) && stan::length(N) && stan::length(theta)))

262  return 1.0;

263

264  double P(1.0);

265

266  // Validate arguments

267  check\_nonnegative(function, N, "Population size parameter", &P);

268  check\_finite(function, theta, "Probability parameter", &P);

269  check\_bounded(function, theta, 0.0, 1.0,

270  "Probability parameter", &P);

271  check\_consistent\_sizes(function, n, N, theta,

272  "Successes variable", "Population size parameter",

273  "Probability parameter",

274  &P);

275

276  // Wrap arguments in vector views

277  VectorView<const T\_n> n\_vec(n);

278  VectorView<const T\_N> N\_vec(N);

279  VectorView<const T\_prob> theta\_vec(theta);

280  size\_t size = max\_size(n, N, theta);

281

282  // Compute vectorized CDF and gradient

283  using stan::math::value\_of;

284  using boost::math::ibeta;

285  using boost::math::ibeta\_derivative;

286

287  agrad::OperandsAndPartials<T\_prob> operands\_and\_partials(theta);

288

289  // Explicit return for extreme values

290  // The gradients are technically ill-defined, but treated as zero

291  for (size\_t i = 0; i < stan::length(n); i++) {

292  if (value\_of(n\_vec[i]) < 0)

293  return operands\_and\_partials.to\_var(0.0);

294  }

295

296  for (size\_t i = 0; i < size; i++) {

297

298  // Explicit results for extreme values

299  // The gradients are technically ill-defined, but treated as zero

300  if (value\_of(n\_vec[i]) >= value\_of(N\_vec[i])) {

301  continue;

302  }

303

304  const double n\_dbl = value\_of(n\_vec[i]);

305  const double N\_dbl = value\_of(N\_vec[i]);

306  const double theta\_dbl = value\_of(theta\_vec[i]);

307

308  const double Pi = ibeta(N\_dbl - n\_dbl, n\_dbl + 1, 1 - theta\_dbl);

309

310  P \*= Pi;

311

312  if (!is\_constant\_struct<T\_prob>::value)

313  operands\_and\_partials.d\_x1[i]

314  += - ibeta\_derivative(N\_dbl - n\_dbl, n\_dbl + 1, 1 - theta\_dbl) / Pi;

315

316

317  }

318

319  if (!is\_constant\_struct<T\_prob>::value) {

320  for(size\_t i = 0; i < stan::length(theta); ++i) operands\_and\_partials.d\_x1[i] \*= P;

321  }

322

323  return operands\_and\_partials.to\_var(P);

324

325  }

326

327  template <typename T\_n, typename T\_N, typename T\_prob>

328  typename return\_type<T\_prob>::type

329  binomial\_cdf\_log(const T\_n& n, const T\_N& N, const T\_prob& theta) {

330

331  static const char\* function = "stan::prob::binomial\_cdf\_log(%1%)";

332

333  using stan::math::check\_finite;

334  using stan::math::check\_bounded;

335  using stan::math::check\_nonnegative;

336  using stan::math::value\_of;

337  using stan::math::check\_consistent\_sizes;

338  using stan::prob::include\_summand;

339

340  // Ensure non-zero arguments lenghts

341  if (!(stan::length(n) && stan::length(N) && stan::length(theta)))

342  return 0.0;

343

344  double P(0.0);

345

346  // Validate arguments

347  check\_nonnegative(function, N, "Population size parameter", &P);

348  check\_finite(function, theta, "Probability parameter", &P);

349  check\_bounded(function, theta, 0.0, 1.0,

350  "Probability parameter", &P);

351  check\_consistent\_sizes(function, n, N, theta,

352  "Successes variable", "Population size parameter",

353  "Probability parameter",

354  &P);

355

356  // Wrap arguments in vector views

357  VectorView<const T\_n> n\_vec(n);

358  VectorView<const T\_N> N\_vec(N);

359  VectorView<const T\_prob> theta\_vec(theta);

360  size\_t size = max\_size(n, N, theta);

361

362  // Compute vectorized cdf\_log and gradient

363  using stan::math::value\_of;

364  using boost::math::ibeta;

365  using boost::math::ibeta\_derivative;

366

367  agrad::OperandsAndPartials<T\_prob> operands\_and\_partials(theta);

368

369  // Explicit return for extreme values

370  // The gradients are technically ill-defined, but treated as negative infinity

371  for (size\_t i = 0; i < stan::length(n); i++) {

372  if (value\_of(n\_vec[i]) < 0)

373  return operands\_and\_partials.to\_var(stan::math::negative\_infinity());

374  }

375

376  for (size\_t i = 0; i < size; i++) {

377  // Explicit results for extreme values

378  // The gradients are technically ill-defined, but treated as zero

379  if (value\_of(n\_vec[i]) >= value\_of(N\_vec[i])) {

380  continue;

381  }

382  const double n\_dbl = value\_of(n\_vec[i]);

383  const double N\_dbl = value\_of(N\_vec[i]);

384  const double theta\_dbl = value\_of(theta\_vec[i]);

385  const double Pi = ibeta(N\_dbl - n\_dbl, n\_dbl + 1, 1 - theta\_dbl);

386

387  P += log(Pi);

388

389  if (!is\_constant\_struct<T\_prob>::value)

390  operands\_and\_partials.d\_x1[i]

391  += - ibeta\_derivative(N\_dbl - n\_dbl, n\_dbl + 1, 1 - theta\_dbl) / Pi;

392  }

393

394  return operands\_and\_partials.to\_var(P);

395  }

396

397  template <typename T\_n, typename T\_N, typename T\_prob>

398  typename return\_type<T\_prob>::type

399  binomial\_ccdf\_log(const T\_n& n, const T\_N& N, const T\_prob& theta) {

400

401  static const char\* function = "stan::prob::binomial\_ccdf\_log(%1%)";

402

403  using stan::math::check\_finite;

404  using stan::math::check\_bounded;

405  using stan::math::check\_nonnegative;

406  using stan::math::value\_of;

407  using stan::math::check\_consistent\_sizes;

408  using stan::prob::include\_summand;

409

410  // Ensure non-zero arguments lenghts

411  if (!(stan::length(n) && stan::length(N) && stan::length(theta)))

412  return 0.0;

413

414  double P(0.0);

415

416  // Validate arguments

417  check\_nonnegative(function, N, "Population size parameter", &P);

418  check\_finite(function, theta, "Probability parameter", &P);

419  check\_bounded(function, theta, 0.0, 1.0,

420  "Probability parameter", &P);

421  check\_consistent\_sizes(function, n, N, theta,

422  "Successes variable", "Population size parameter",

423  "Probability parameter",

424  &P);

425

426  // Wrap arguments in vector views

427  VectorView<const T\_n> n\_vec(n);

428  VectorView<const T\_N> N\_vec(N);

429  VectorView<const T\_prob> theta\_vec(theta);

430  size\_t size = max\_size(n, N, theta);

431

432  // Compute vectorized cdf\_log and gradient

433  using stan::math::value\_of;

434  using boost::math::ibeta;

435  using boost::math::ibeta\_derivative;

436

437  agrad::OperandsAndPartials<T\_prob> operands\_and\_partials(theta);

438

439  // Explicit return for extreme values

440  // The gradients are technically ill-defined, but treated as negative infinity

441  for (size\_t i = 0; i < stan::length(n); i++) {

442  if (value\_of(n\_vec[i]) < 0)

443  return operands\_and\_partials.to\_var(0.0);

444  }

445

446  for (size\_t i = 0; i < size; i++) {

447  // Explicit results for extreme values

448  // The gradients are technically ill-defined, but treated as zero

449  if (value\_of(n\_vec[i]) >= value\_of(N\_vec[i])) {

450  return operands\_and\_partials.to\_var(stan::math::negative\_infinity());

451  }

452  const double n\_dbl = value\_of(n\_vec[i]);

453  const double N\_dbl = value\_of(N\_vec[i]);

454  const double theta\_dbl = value\_of(theta\_vec[i]);

455  const double Pi = 1.0 - ibeta(N\_dbl - n\_dbl, n\_dbl + 1, 1 - theta\_dbl);

456

457  P += log(Pi);

458

459  if (!is\_constant\_struct<T\_prob>::value)

460  operands\_and\_partials.d\_x1[i]

461  += ibeta\_derivative(N\_dbl - n\_dbl, n\_dbl + 1, 1 - theta\_dbl) / Pi;

462  }

463

464  return operands\_and\_partials.to\_var(P);

465  }

466

467

468  template <class RNG>

469  inline int

470  binomial\_rng(const int N,

471  const double theta,

472  RNG& rng) {

473  using boost::variate\_generator;

474  using boost::binomial\_distribution;

475

476  static const char\* function = "stan::prob::binomial\_rng(%1%)";

477

478  using stan::math::check\_finite;

479  using stan::math::check\_less\_or\_equal;

480  using stan::math::check\_greater\_or\_equal;

481  using stan::math::check\_nonnegative;

482

483  check\_nonnegative(function, N,

484  "Population size parameter", (double\*)0);

485  check\_finite(function, theta,

486  "Probability parameter", (double\*)0);

487  check\_less\_or\_equal(function, theta, 1.0,

488  "Probability parameter", (double\*)0);

489  check\_greater\_or\_equal(function, theta, 0.0,

490  "Probability parameter", (double\*)0);

491

492  variate\_generator<RNG&, binomial\_distribution<> >

493  binomial\_rng(rng, binomial\_distribution<>(N, theta));

494  return binomial\_rng();

495  }

496

497  }

498 }

499 #endif

stan::agrad::log1m

fvar< T > log1m(const fvar< T > &x)

**Definition:** log1m.hpp:16

multiply\_log.hpp

stan::math::check\_bounded

bool check\_bounded(const char \*function, const T\_y &y, const T\_low &low, const T\_high &high, const char \*name, T\_result \*result)

**Definition:** check\_bounded.hpp:79

traits.hpp

stan::agrad::OperandsAndPartials::to\_var

T\_return\_type to\_var(double logp)

**Definition:** partials\_vari.hpp:138

stan::prob::binomial\_cdf\_log

return\_type< T\_prob >::type binomial\_cdf\_log(const T\_n &n, const T\_N &N, const T\_prob &theta)

**Definition:** binomial.hpp:329

error\_handling.hpp

stan::math::multiply\_log

boost::math::tools::promote\_args< T\_a, T\_b >::type multiply\_log(const T\_a a, const T\_b b)

Calculated the value of the first argument times log of the second argument while behaving properly w...

**Definition:** multiply\_log.hpp:51

stan::math::log\_inv\_logit

boost::math::tools::promote\_args< T >::type log\_inv\_logit(const T &u)

Returns the natural logarithm of the inverse logit of the specified argument.

**Definition:** log\_inv\_logit.hpp:36

stan::length

size\_t length(const T &)

**Definition:** traits.hpp:159

stan::math::binomial\_coefficient\_log

boost::math::tools::promote\_args< T\_N, T\_n >::type binomial\_coefficient\_log(const T\_N N, const T\_n n)

Return the log of the binomial coefficient for the specified arguments.

**Definition:** binomial\_coefficient\_log.hpp:63

stan::DoubleVectorView

DoubleVectorView allocates double values to be used as intermediate values.

**Definition:** traits.hpp:358

stan::math::check\_finite

bool check\_finite(const char \*function, const T\_y &y, const char \*name, T\_result \*result)

Checks if the variable y is finite.

**Definition:** check\_finite.hpp:52

stan::prob::binomial\_log

return\_type< T\_prob >::type binomial\_log(const T\_n &n, const T\_N &N, const T\_prob &theta)

**Definition:** binomial.hpp:31

stan::math::inv\_logit

boost::math::tools::promote\_args< T >::type inv\_logit(const T a)

Returns the inverse logit function applied to the argument.

**Definition:** inv\_logit.hpp:52

partials\_vari.hpp

stan::agrad::value\_of

T value\_of(const fvar< T > &v)

Return the value of the specified variable.

**Definition:** value\_of.hpp:16

constants.hpp

stan::prob::binomial\_rng

int binomial\_rng(const int N, const double theta, RNG &rng)

**Definition:** binomial.hpp:470

stan::agrad::OperandsAndPartials

A variable implementation that stores operands and derivatives with respect to the variable...

**Definition:** partials\_vari.hpp:76

traits.hpp

stan::agrad::inv\_logit

fvar< T > inv\_logit(const fvar< T > &x)

**Definition:** inv\_logit.hpp:15

stan::return\_type::type

boost::math::tools::promote\_args< typename scalar\_type< T1 >::type, typename scalar\_type< T2 >::type, typename scalar\_type< T3 >::type, typename scalar\_type< T4 >::type, typename scalar\_type< T5 >::type, typename scalar\_type< T6 >::type >::type type

**Definition:** traits.hpp:406

stan::is\_constant\_struct

Metaprogram to determine if a type has a base scalar type that can be assigned to type double...

**Definition:** traits.hpp:57

stan::math::value\_of

double value\_of(const T x)

Return the value of the specified scalar argument converted to a double value.

**Definition:** value\_of.hpp:24

stan::prob::include\_summand

Template metaprogram to calculate whether a summand needs to be included in a proportional (log) prob...

**Definition:** traits.hpp:35

log1m.hpp

stan::agrad::ibeta

var ibeta(const var &a, const var &b, const var &x)

The normalized incomplete beta function of a, b, and x.

**Definition:** ibeta.hpp:223

stan::math::check\_nonnegative

bool check\_nonnegative(const char \*function, const T\_y &y, const char \*name, T\_result \*result)

**Definition:** check\_nonnegative.hpp:52

log\_inv\_logit.hpp

stan::math::check\_consistent\_sizes

bool check\_consistent\_sizes(const char \*function, const T1 &x1, const T2 &x2, const char \*name1, const char \*name2, T\_result \*result)

**Definition:** check\_consistent\_sizes.hpp:13

stan::max\_size

size\_t max\_size(const T1 &x1, const T2 &x2)

**Definition:** traits.hpp:191

stan::prob::binomial\_ccdf\_log

return\_type< T\_prob >::type binomial\_ccdf\_log(const T\_n &n, const T\_N &N, const T\_prob &theta)

**Definition:** binomial.hpp:399

stan::math::check\_less\_or\_equal

bool check\_less\_or\_equal(const char \*function, const T\_y &y, const T\_high &high, const char \*name, T\_result \*result)

**Definition:** check\_less\_or\_equal.hpp:52

stan::agrad::multiply\_log

fvar< T > multiply\_log(const fvar< T > &x1, const fvar< T > &x2)

**Definition:** multiply\_log.hpp:15

stan::math::size

int size(const std::vector< T > &x)

**Definition:** size.hpp:11

stan::agrad::binomial\_coefficient\_log

fvar< T > binomial\_coefficient\_log(const fvar< T > &x1, const fvar< T > &x2)

**Definition:** binomial\_coefficient\_log.hpp:16

value\_of.hpp

stan::agrad::log\_inv\_logit

fvar< T > log\_inv\_logit(const fvar< T > &x)

**Definition:** log\_inv\_logit.hpp:15

inv\_logit.hpp

stan::agrad::OperandsAndPartials::d\_x1

VectorView< double \*, is\_vector< T1 >::value, is\_constant\_struct< T1 >::value > d\_x1

**Definition:** partials\_vari.hpp:82

binomial\_coefficient\_log.hpp

constants.hpp

stan::prob::binomial\_cdf

return\_type< T\_prob >::type binomial\_cdf(const T\_n &n, const T\_N &N, const T\_prob &theta)

**Definition:** binomial.hpp:249

stan::agrad::log

fvar< T > log(const fvar< T > &x)

**Definition:** log.hpp:15

stan::VectorView

VectorView is a template metaprogram that takes its argument and allows it to be used like a vector...

**Definition:** traits.hpp:275

stan::math::check\_greater\_or\_equal

bool check\_greater\_or\_equal(const char \*function, const T\_y &y, const T\_low &low, const char \*name, T\_result \*result)

**Definition:** check\_greater\_or\_equal.hpp:58

stan::math::log1m

boost::math::tools::promote\_args< T >::type log1m(T x)

Return the natural logarithm of one minus the specified value.

**Definition:** log1m.hpp:40

stan::prob::binomial\_logit\_log

return\_type< T\_prob >::type binomial\_logit\_log(const T\_n &n, const T\_N &N, const T\_prob &alpha)

**Definition:** binomial.hpp:142

stan::math::negative\_infinity

double negative\_infinity()

Return negative infinity.

**Definition:** constants.hpp:123


---

[ Stan Home Page ]

*© 2011–2014,
Stan Development Team.*
